# A Computational Analysis of Diagnostic Misrecognition in Co-Occurring Primary Headache Disorders: Prime Encoding of ICHD3 Criteria Reveals 72 Potentially Spurious Diagnoses

**DOI:** 10.3390/diagnostics16193231

**Published:** 2026-10-07

**Authors:** Pengfei Zhang

**Affiliations:** 1 Department of Neurology, Beth Israel Deaconess Medical Center, Boston, MA 02215, USA; pzhang7@bidmc.harvard.edu; 2 Harvard Medical School, Boston, MA 02215, USA

**Keywords:** headache classifications, mathematical models, misdiagnosis, migraine

## Abstract

**Background/Objectives**: In clinical circumstances with more than one headache disorder, misrecognition can occur if each headache disorder is not separately and individually evaluated. For example, consider the case of a patient with baseline tension-type headaches lasting hours who now develops cluster headaches with nausea. Merging the characteristics of both disorders may yield the illusion of migraine. Do these types of misrecognitions occur necessarily because of ICHD3 criteria? Are there combinations of ICHD3 criteria, if any, which yield this type of misrecognition? This work seeks to determine whether a new, unintended diagnosis can be arrived at when one merges two primary ICHD3 diagnoses. **Methods**: ICHD3 primary headache disorders, as well as headache phenotypes closely mimicking ICHD3 criteria, were translated as numerical data using the prime encoding method. The encoding of two concurrent diagnoses in an individual was then modeled by multiplying all pairings of the ICHD3 encoding. The results were algorithmically diagnosed to see which pairings, if any, generate logically spurious diagnoses. **Results**: A total of 117,526 phenotypes were generated. After duplicate pairings of diagnoses were removed, a total of 72 pairings of unique ICHD3 diagnoses were derived that yield a “new” diagnosis. Thirty-seven (51%) of these pairings arise from our model’s idiosyncrasy of encoding chronic migraine without encoding migraine with or without aura explicitly. Of the rest, 23 pairings involve combining paroxysmal hemicrania with either a longer- or shorter-duration headache. I further generalize the condition under which the above logically spurious diagnosis occurs as a mathematical observation. **Conclusions**: Misrecognition arises naturally if diagnostic criteria consist solely of characteristics from another headache disorder or a combination of two headache disorders. Specifically, combining headaches with different duration and/or frequency is most likely to yield these “logically spurious” diagnoses.

## 1. Introduction

Co-occurrences of multiple headache disorders may lead to misdiagnosis if each is not individually evaluated. Consider, for example, a patient with baseline tension-type headaches (TTHs) developing a new cluster headache (CH) with nausea: the combination of symptoms—headache lasting 30 min to 7 days (from tension-type headaches), unilaterality (from new cluster headaches), and association with nausea (from this particular clinical presentation)—may appears to satisfy migraine criteria [1]. In this example, nausea is extrinsic to the diagnostic criteria of TTH and CH. However, this example motivates a crucial question: are there pairings of headache disorders in the International Classifications of Headache Disorders (ICHD3), when occurring simultaneously, that lead to a “logically spurious” third diagnosis? This problem is important for clinical practice and research: if these “misrecognitions” arise whenever two specific disorders occur concurrently, then an increased likelihood for misdiagnosis would be built into ICHD3, leading to erroneous inclusion or exclusion of patients in clinical trials and incorrect treatment in the clinical encounters.

This work provides an answer for these questions of misrecognition in ICHD3. By enumerating and merging all possible pairings of diagnoses from the ICHD3, I determined which, if any, of these pairings yield a third “illusionary” diagnosis—I will term these “logically spurious” diagnoses; the term ‘illusionary’ will be used only in circumstances to evoke the clinical phenomenon of misrecognition. Finally, I determined the rules making this sort of misrecognition possible from a classification point of view, providing a framework to identify and/or avoid them in future classifications.

## 2. Materials and Methods

To study classification criteria en masse, one needs to translate ICHD3 into manipulable numerical data. In prior papers, I demonstrated that it is possible to do so through the following [2,3]: First, one represents every headache characteristic, whether positive criteria (i.e., photophobia, phonophobia, nausea, etc.) or negative criteria (i.e., no photophobia, no phonophobia, no nausea, etc.), as a prime number. One then represents every headache phenotype within a headache diagnosis as composite numbers by an encoding algorithm:

1. Translate headache criteria into a series of logical AND statements connected by OR statements (This is called “disjunctive normal form” and can be done for all Boolean logic statements).
2. For every AND that is used between two characteristics, the corresponding prime numbers for those two characteristics are multiplied together. The results are called encodings.
3. All composite numbers that are generated, and separated by an OR statement, are then combined into a list.

Please note that the criterion “not attributed to another disorder” is not modeled in our encoding due to potential introduction of logical impasse (see Section 4).

The above is feasible due to the connection between logical statements and prime number composition (In fact, this encoding method is merely a variant of Gödel numbering [4]. Furthermore, this method is equivalent to a matrix representation of ICHD3 [5]). I will briefly describe the above algorithm through an example but refer the interested reader to the original article for more information (The complete list of encodings as well as prime number representations is presented in our prior articles [2]. For the ease of the reader, prime representations are reproduced in Supplementary File S1 in the Supplemental Materials of this text with permission. Mathematical proof of this method is also available in the Supplemental Material of the prior article, which in turn was adopted from a more comprehensive preprint [2,3]).

### 2.1. Example of an Encoding

Consider the ICHD3 criteria for cluster headache; one can represent all possible phenotypes of the disorder as the following series of composite numbers.

1577982193416431, 1932584933510011, 2251727399594233, 3492836989921763, 4237502744118281, 4911247950296083, 7428927404960501, **7960831515100871**, 8173593159157019

Similarly, for infrequent tension type headaches:

1378253359942681, 1386197183342927, 2206388426002661, 2219105362175587, 2241879928833803, **2254801426982701**, 9699783080351321, 9755689611073807, 9855811762608983, 9912617594093761, 15777758744434123, 15868696835180141, 514088503258620013, 517051549386911771, 522358023418276099, 525368732486969333, 836221213455008519, 841040932264547473, 3676217787453150659, 3697406362596972853, 194839542735016984927, 195962537217639561209

Important to our discussion is that the encoding algorithm contains the ability for automated diagnosis through modular arithmetic: when the phenotype of a specific patient’s headache profile, as encoded by a prime number, can be divided by the encoding of a diagnosis without remainder, then that diagnosis is satisfied according to the ICHD3. For the sake of this manuscript, I will call the former encoding “target clinical encoding” and the latter encoding “diagnostic encoding”.

For example, consider the patient with both infrequent tension-type headaches and episodic cluster headaches, the autonomic feature being restlessness, with symptoms of nausea. The set of characteristics, as well as its associated prime encoding, is the following according to the ICHD3: nausea (281), greater than 5 episodes (157), severe (479), unilateral (523), orbital or supraorbital (383), duration between 15 min and 3 hours (11), restlessness (449), one every other day to 8 per day (107), >10 episodes of headaches (257), less than 12 days per year (199), 30 min to 7 days (19), bilateral (53), not aggravated by routine activities (379), no nausea (331), no photophobia (349), and no phonophobia (347).

(Notice that in this specific case the patient would describe “no nausea” for his tension-type headaches but with nausea for his new onset of cluster headaches).

Multiplying the above sequences of prime encoding would generate the following composite number:

1750259841031105929758798277984900497

This number is the target clinical encoding representing this clinical scenario. Notice that this number is divisible by 7960831515100871 and 2254801426982701 without remainder. The former of these two numbers is a diagnostic encoding for the criteria of cluster headaches, whereas the latter is a diagnostic encoding for tension-type headaches (these are bolded in the encodings above). Therefore, this patient has a diagnosis of cluster headache and infrequent tension-type headache. This example is not an isolated occurrence; rather, it can be proven that given a set of headache characteristics, the above algorithm would work for automated diagnosis of any ICHD3 headache disorders [2,3].

### 2.2. General Paradigm of the Method

Generalizing from the above, the method of investigating concurrent diagnosis essentially involves the following:

1. I generate a list of all possible phenotypes for each headache diagnosis.
2. I multiply all possible combinations of step 1 to model two concurrent phenotypes in a single clinical scenario as target clinical encodings. This is simply a generalization of the example above in Section 2.1.
3. I then use our automated diagnostic technique to diagnose each of the target clinical encodings generated in step 2. Again, notice that this automated diagnostic technique also uses prime encoding, called the diagnostic encoding (see example in Section 2.1). The differentiation between target clinical encoding and diagnostic encoding will be important to our discussion below.

### 2.3. Modification to the Encoding to Account for Implications

Notice that in our example above (Section 2.1), encoding cannot be divisible by any of the encodings for migraine without aura and so migraine is not a diagnosis for the above combinations of characteristics. The reason is that although a headache that lasts “30 min to 7 days” (19) could also be a headache that lasts between “4 h to 72 h” (23), a migraine criterion, the two characteristics are encoded separately in the original database.

To account for these kinds of implications, the target clinical encoding must not only include characteristics that are verbatim in the classification criteria but also those other characteristics that are implied by the criteria logically. This was not accounted for in the prior manuscript.

In order to adjust for this, I first manually compiled a list of characteristics which implies or could imply the other in Table 1. For example, the criterion “15 min to 180 min” (11) may imply the criterion “30 min to 7 days in duration” (19), since some duration that satisfies the former may also satisfy the latter (in other words, elements of the former criterion may be subset of the latter). This is therefore included in Table 1. On the other hand, a headache that lasts 170 min can be thought of as having the duration of hours; therefore, the criterion “15 min to 180 min” (11) is also an implication of “hours to days” (179) (in other words, there exists an element in the former criterion which is also in the latter criterion). The decision to include the latter stems from the need to capture and model ambiguity in clinical circumstances such as those presented in our motivating example.

Once Table 1 is generated, any encoding that contains a characteristic in column 1 of Table 1 is then multiplied by a prime number representing the implied characteristics in column 2: similar to our example in Section 2.1, this process artificially adds the implied criterion to the target clinical encoding (Multiplication works in this setting since the presence of multiple concurrent characteristics can be represented by the logic AND in the target clinical encoding. Notice that we did not change diagnostic encoding). For example, a phenotype which had previously only contains “up to a few second (557)” now also contains “1 to 600 seconds (5)”, “1 second to 2 hours (47)”, and “less than 48 hours (211)”. So as a result, when automated diagnosis is applied via modular arithmetic through the unchanged diagnostic encoding, the new number can now be divided, without remainder, by any related diagnostic criteria.

The result is therefore a new numerical database of clinical circumstances (i.e., target clinical encodings) representing all possible phenotypes of headache diagnoses as well as close mimics of each characteristic (again, the new encoding is done for target clinical encoding and not for diagnostic encoding). When needed to differentiate this from all other encodings, we will call these the “modified encodings”. This terminology will be used for the proof below.

Given this revised list, I then calculate the combination of any pairing of two headache phenotypes by multiplying each phenotypical modified encoding with all other modified phenotypical encoding. Doing so models two phenotypes presenting concurrently in a single patient as our target clinical encoding of interest for this project (similar to our numerical example above). Keeping track of which disorders I am merging, I then applied our aforementioned automated diagnosis technique (through the unmodified, original, ICHD3 encoding, i.e., diagnostic encoding) to the multiplied list.

In summary, the following modifications were made to the data from the previous manuscript:

1. Manual generation of Table 1 to determine which characteristics imply or may imply the other.
2. Algorithmically going through each of the original prime encoding phenotypes in the prior paper; for each characteristic (i.e., each unique prime listed in column 1 of Table 1), multiply that phenotype with those of column 2.
3. Each modified encoding generated by the last step is then multiplied by all other modified encodings.
4. Automated diagnosis is applied through modular arithmetic via the unchanged diagnostic encoding. The input of the last step is tracked algorithmically.

As a result of the above modification, the target clinical encoding generated here differs from the encoding used in the previous paper to detect concurrent diagnosis by allowing for illogical characteristics. In the previous paper, the question is whether one headache phenotype can produce two equally valid ICHD3 diagnoses; therefore, imposing constraints of internal logical consistency of the target clinical encoding is needed [2]. In contrast, this paper asks the opposite: whether combining two separate headache phenotypes—which may have logically conflicting characteristics with each other—can generate additional spurious phenotype(s). As a result, a total of 117,526 targeted clinical encodings were generated as opposed to 253,842 encodings [2] (see Section 3). Furthermore, since the new data is modified to include additional characteristics from Table 1, each target clinical encoding is also different from the prior paper.

All the above algorithms are generated and implemented through custom codes in Haskell version 9.4.8 and are included in the Supplementary Materials.

## 3. Results

Twenty-seven logical implication statements were obtained (see Table 1) and the encoding database was modified accordingly (please see Section 2.2 above for an itemized list). Using the new modified encoding data, a total of 117,526 phenotypes were generated through merging pairings of headache phenotypes. Each was then diagnosed through automated diagnosis.

Duplicates—defined as multiple different kinds of pairing phenotypes producing the same final combination of diagnosis—are possible in our methodology. For example, the pairing of a cluster headache with restlessness and migraine with visual aura has the same diagnosis as cluster headache with lacrimation and migraine with sensory aura. Since both are migraine with aura and cluster, they are considered duplicates. These duplicates are removed.

Table 2 represents a complete table of 72 pairings in which a new logically spurious diagnosis is generated. Notice that in the above results, some of the diagnoses are not actually logically spurious but rather contain artifacts from our model’s idiosyncrasy of encoding chronic migraine: in the ICHD3, chronic migraine is required to be either migraine with aura or migraine without aura. As a result, the encoding for chronic migraine always includes within it encoding for migraine with aura or without aura. Therefore, whenever chronic migraine is merged with another diagnosis, inevitably the result would include an additional diagnosis of migraine without aura or migraine with aura. There are 37 such results, or 51.1% of the 72 pairings. Those that are not caused by this idiosyncrasy are highlighted with an asterisk in Table 2.

A more clinically useful table with these idiosyncrasies removed is also presented in Table 3. Notably, I considered the first two combinations—the generation of *frequent* tension-type headaches as a spurious diagnosis when combining chronic migraine with and without aura with *chronic* tension-type headaches—to be trivial from a clinical perspective. These two are therefore not included in Table 3. The occurrences of these two arise out of the 8-day rule (criteria C) in the chronic migraine definition; this rule allows for an appearance of a logically spurious 8-day tension-type headache leading, i.e., the spurious frequent tension-type headache.

Two pairings in the result yield 5 concurrent headache diagnoses. Both of these arise from the combination of paroxysmal hemicrania and chronic migraine. Part of the reason is from the idiosyncrasy of chronic migraine encoding, as discussed above. However, combining paroxysmal hemicrania and chronic migraine can still produce the illusion of cluster and SUNCT. The reason for this is two-fold: 1. Since SUNCT is defined as between 1 and 600 seconds and PH between 2 minutes and 30 minutes, there is an overlap in timeframe of these diagnoses. Since SUNCT is not defined as indomethacin non-responsive, this allows for concurrent diagnoses of both. 2. PH and cluster headache duration also overlap. As a result, when PH is merged with chronic migraine criteria, a headache that is at least 15 days per month, the criteria of frequency for cluster headache are also satisfied, allowing for a diagnosis of the latter. In other words, CM criteria lead to the illusion of longer and more frequent headaches of the TAC phenotype, distorting the original diagnosis that can lead to two more potential headaches. Clinically, the above observations may support a diagnostic strategy of trying indomethacin in patients whom one suspects to have cluster or SUNCT/SUNA, even though neither of these diagnoses should be indomethacin-responsive; the rationale is that successful indomethacin trials in these cases may clarify whether any misrecognition has occurred in history taking.

Indeed, PH, when merging with another headache of different duration and frequency, allows for distortion, and therefore, the illusion of a new diagnosis can be seen in 23 of the pairings in Table 2. Specifically, combining PH with a shorter-lasting headache—such as primary sex headaches, cough headaches, primary stabbing headaches, exercise headaches, traction headache—leads to the logically spurious diagnosis of SUNCT. This is logical since one can interpret the short duration to be associated with the TAC features. Combining PH with a longer-lasting headache—such as new daily persistent headache—leads to the illusion of hemicrania continua. This pattern of “time-distortion” also goes the opposite direction: combining a long-lasting headache—such as chronic tension-type headache or HC—with a very short-lasting headache—such as SUNCT—produces a medium-duration headache such as cluster or PH as an illusion. Therefore, in addition to considering an indomethacin trial on suspected SUNCT/SUNA or cluster, a case can be made for tracking durations and frequency of attacks with TAC features, as opposed to just duration of all attacks.

Finally, clinicians are most likely to encounter migraine as well as tension-type headaches, given the prevalence of these primary headache disorders [6,7,8]. As a result, I have extracted from Table 3 the pairing containing migraine and TTH (Table 4) as an aid to clinicians. I have also included examples of these co-occurring phenotypes in the Supplemental Material. Derived from these Supplemental Materials, and included in Table 4, I have also noted specific features of these pairings which may hint to clinicians the possibility of co-occurrence.

### 3.1. Generalization

It would be beneficial to determine the condition of possibility in which logically spurious diagnosis occurs, allowing for a way to construct classification guidelines so as to avoid these pairings. This is possible by considering diagnostic criteria as prime number encodings; I present the following theorem as well as its mathematical proof.

**Theorem** **1.**
*Given two different modified diagnosis encodings, p and q, let d be the encoding of a logically spurious diagnosis that arises from the combination of both. Then d exists (i.e., d does not equal p nor q) only if its characteristics are some combination of the characteristics of p and q or their subsets.*

**Proof.**  Let *p* = *p*_1_*p*_2_ … *p_i_* … *p_n_* and *q = q*_1_*q*_2_ *… q_i_ … q_m_* be modified encodings where each *p_i_* and *q_i_* are unique prime encoding of characteristics. Assuming that *d* exists, then it must divide *p* multiply by *q* without remainder. Then *d* must divide the product of some combination of *p_i_* and *q_i_* without remainder. Since all *p_i_* and *q_i_* are prime numbers and, by definition, *d* does not equal *p* nor *q*, then *d* must either: 1. divide *p* without remainder and therefore is the product of some subset of *p_i_*; 2. divide *q* without remainder and therefore is the product of some subset *q_i_*; 3. equal the product of some combination of subsets of *p_i_* and *q_i_*. □

The proof above arises naturally from our construction of prime number encoding as well as its ability to automate diagnoses. Furthermore, it suggests that one can avoid logically spurious diagnosis so long as any new headache disorder—that is, *d*, in our proof—does not contain solely the characteristics, nor related characteristics, such as those in Table 1, to another pairing of headache diagnoses.

### 3.2. Application to Other Headache Disorders

Let us see how one can apply the above theorem to three examples of secondary headache disorders—CSF leak (i.e., headache attributed to low CSF pressure), medication overuse headache (MOH), and persistent post-traumatic headache (PTH).

First, let us consider the ICHD3 definition of headache attributed to low CSF pressure [1]:

- Any headache fulfilling criterion C.
- Either or both of the following:
  - low cerebrospinal fluid (CSF) pressure (<60 mm CSF);
  - evidence of CSF leakage on imaging.
- Headache has developed in temporal relation to the low CSF pressure or CSF leakage, or led to its discovery.
- Not better accounted for by another ICHD-3 diagnosis.

In accordance with the method of this manuscript, we will not consider criterion D. Notice that criterion A simply refers to criterion C; it is therefore sufficient to consider characteristics derived from criteria B and C only. Now the components of criteria B and C are actually not repeated anywhere else in the ICHD3 (aside from the subtypes of CSF leak). As a result, CSF leak criteria cannot form a subset with other headache disorders according to our theorem. Logically spurious diagnosis therefore does not form organically from this criterion.

Now consider the definition of medication overuse headaches (MOH) [1]:

- Headache occurring on greater than or equal to 15 days/month in a patient with a pre-existing headache disorder.
- Regular overuse for >3 months of one or more drugs that can be taken for acute and/or symptomatic treatment of headache.
- Not better accounted for by another ICHD-3 diagnosis.

In accordance with the method of this manuscript, we will again discard criterion C. As a result, criterion A, criterion B, and MOH itself can be thought of as the variables *p*_1_, *p*_2_, and *p*, in our proof, respectively. Whether a spurious diagnosis exists depends on whether we can find another primary or secondary diagnosis, let us call it *q*, from ICHD3 such that some component of its criteria *q*_1_*, q*_2_*, … q_n_* together with *p*_1_ and *p*_2_ may form a superset of some diagnosis, i.e., a diagnosis *d*.

Notice that *p*_1_ definitionally includes any other headaches with more than 15 headache days. Furthermore, criteria *p*_2_ is not found anywhere else except for subsections of MOH. These two observations suggest that any potential logically spurious diagnosis, *d*, if it exists, must therefore contain the criterion of “more than 15 days per month”. Since the further requirement is that *d* cannot be *q*—we may arrive at *d* by simply searching for a diagnosis in the classification where, while containing the phrase “more than 15 days per month”, it also contains criteria from a third unrelated diagnosis. Indeed, a search of the phrase “15 days” in ICHD3 yields one such result—sleep apnea headache [1]:

- Headache present on awakening after sleep and fulfilling criterion C.
- Sleep apnea where apnea–hypopnea index has been diagnosed.
- Evidence of causation demonstrated by at least two of the following:
  1. headache has developed in temporal relation to the onset of sleep apnea;
  2. either or both of the following:
    (a) headache has worsened in parallel with worsening of sleep apnea;
    (b) headache has significantly improved or remitted in parallel with improvement in or resolution of sleep apnea;
  3. headache has at least one of the following three characteristics:
    (a) recurring on greater than or equal to 15 days/month;
    (b) all of the following:
      - bilateral location;
      - pressing quality;
      - not accompanied by nausea, photophobia or phonophobia;
    (c) resolving within four hours.
- Not better accounted for by another ICHD-3.
- Diagnosis.

Notice that criteria C3*a*, C3*b,* and C3*c* are equivalent to a diagnosis of chronic tension-type headache. As a result, chronic TTH and sleep apnea headache satisfies *d* and *q* in our theorem, respectively. The combination of sleep apnea headache and medication overuse headache therefore can yield the logically spurious diagnosis of chronic tension-type headache.

Finally, consider the case of persistent PTH, or persistent headache attributed to traumatic injury to the head [1]:

- Any headache fulfilling criteria C and D.
- Traumatic injury to the head has occurred.
- Headache is reported to have developed within seven days after one of the following:
  1. the injury to the head;
  2. regaining of consciousness following the injury to the head;
  3. discontinuation of medication(s) impairing ability to sense or report headache following.
- The injury to the head.
- Headache persists for >3 months after its onset.
- Not better accounted for by another ICHD-3 diagnosis.

One applies the logic derived from the theorem in much the same fashion as for MOH. Firstly, one can restrict our search to disorders outside of headache attributed to trauma or injury to the head and/or neck since these criteria were designed to be distinct. Notice that apart from criteria E—“headache persisting for >3 months”—none of the criteria are listed in any other ICHD3 diagnosis. Searching the ICHD3 for this particular phrase, looking for a criterion where the remainder of the criteria would satisfy another diagnosis, yields the obvious choices of PH and HC. Since PH and HC are separated only by frequency and duration, PH occurring concurrently with persistent PTH would lead to a misrecognition of HC; here HC is satisfied by its constancy for more than 3 months via PTH and satisfying its TAC features as well as indomethacin response through PH.

The above 3 examples serve as a demonstration of the application of the above theorem in searching for logically spurious diagnoses beyond primary headaches in the ICHD3. One can use the same logic in conducting similar searches for logically spurious diagnosis in other secondary headaches and facial pain.

### 3.3. Extension to More than Two Diagnoses

More than two diagnoses may concurrently occur in real-life clinical scenarios. The results above can be extended to these cases based the observation that any co-occurrence of 3 headaches necessarily implies co-occurrence of 2 headaches. Let us designate the 3-member co-occurrence headaches by the set {*a*, *b*, *c*}, where *a*, *b*, and *c* are unique headache disorders. This co-occurrence already implies a co-occurrence of {*a*, *b*}*,* {*b*, *c*}, and {*a*, *c*} in the same set. As such, it is sufficient to enumerate all co-occurrences of 2 headaches to explore the co-occurrence of 3 headaches. This is the result of this paper.

For example, consider the case of the co-occurrence of exercise-induced headache, cluster headache, as well as SUNCT. One simply needs to see the output for {exercise-induced headache, cluster headache}, {exercise-induced headache, SUNCT}, and {cluster headache, SUNCT} to see the potentially logically spurious output. Based on Table 2, this would produce outputs of {exercise-induced headache, cluster headache, SUNCT}, {exercise-induced headache, SUNCT}, and {cluster headache, SUNCT}. (Note: the latter 2 are not in Table 2, implying that “logically spurious” diagnoses do not exist for those pairings.) Simplifying this output yields {exercise-induced headache, cluster headache, SUNCT}. Therefore, in the triplet of {exercise-induced headache, cluster headache, SUNCT} there is no logically spurious diagnosis.

Notice that the above equivalence does not always apply. Consider the case of the co-occurrence of {thunderclap headache, PH, traction-induced headache}. This set of {thunderclap headache, PH, traction-induced headache} would be evaluated by looking up {thunderclap headache, PH}, {PH, traction-induced headache}, {thunderclap headache, traction-induced headache} in Table 2. This yields the following outputs, respectively: {thunderclap headache, PH, SUNCT/SUNA}, {traction-induced headache, PH, SUNCT/SUNA}, {thunderclap headache, traction-induced headache}. (The combination of two headaches is, of course, abelian in this setting.) Therefore, the resultant diagnosis—{thunderclap headache, PH, SUNCT, traction-induced headache}—would contain SUNCT/SUNA as a logically spurious diagnosis.

Indeed, any *n*-many co-occurring headaches can be derived from 2 co-occurring headache pairings through the above process. In other words, these 3 co-occurring situations are fundamentally not so different from 2 co-occurring: each circumstance contains a list of real diagnoses as a first assumption but their merging characteristics may or may not produce an additional spurious conclusion. Notice that this paper does not suggest a mathematical way of discovering the spurious diagnosis; rather, I advocate that the correct way to discern genuine diagnoses is through history taking.

## 4. Discussion

Despite being the most common complaint in neurology, headaches disorders are often misdiagnosed and constitute significant public health problems [9,10,11]. This is particularly true in geriatric settings where co-morbidities are common [11]. The reasons for misdiagnosis range from the ambiguity of the presenting disorders, disorders straddling multiple diagnoses, or imprecise history taking; nevertheless, a subset of misdiagnosis arises because multiple concurrent disorders are present [11,12,13,14,15,16]. For example, the conception that cluster headache may present with aura has recently been challenged by Peng et al.: TAC patients with “aura” may be due to comorbid migraine disorder in cluster headache patients [16,17]. Primary headaches often co-occur: consider, for example, that primary stabbing headaches often co-occur in the setting of migraines or other TAC and migraine or tension-type headaches co-occur in the setting of NDPH [18,19,20]. Therefore, the problem of multiple co-existing headaches leading to misdiagnosis is a realistic concern in clinical practice. This article attempts to study these misrecognitions, or what I call “logically spurious” diagnoses, that may arise whenever multiple headache disorders are merged as one.

Two results emerge from this study: 1. Merging headaches with divergent frequency and/or duration is most susceptible to generating misrecognition. This is particularly true if headache triggers—such as cough, sexual activities, etc.—of the so called “other primary headache disorders” are ignored or not recognized during history taking. 2. When phenotypes of diagnostic criteria are subset(s) of other criteria, misrecognition can occur. Clinically, this work highlights the importance of interrogating each co-occurring headache individually in history taking [21]. This is especially important in the diagnosis of TACs; duration of the illness is often the key feature and therefore the misrecognition of divergent coexisting primary headaches may disrupt and distort diagnoses.

Although this paper primarily limits itself to primary headaches, in real-life settings, primary headaches exist alongside secondary headaches, which may further prompt misrecognition. Firstly, secondary headaches may present with a wide range of duration and frequency (in the case of CSF leak, for example, the onset/resolution of headaches varies widely after position change [22]). Therefore, co-existence of primary headaches with secondary headaches of different duration may lead to the perception of logically spurious diagnoses. Secondly, medication overuse headaches (MOHs) have been reported to change the primary headache’s presenting phenotype; co-occurrence of MOH in both primary and secondary headaches further allows for misrecognition [23]. Since the occurrence of MOH already implies the existence of two headache disorders, the clinical situation may rapidly evolve in complexity: consider the patient who has one baseline primary headache disorder who then suffers a common secondary headache—such as post-traumatic headache—and subsequently self-medicates.

The way to overcome misrecognition is through detailed history taking. As championed by Deborah Friedman, this paper highlights the importance of asking about each headache pain individually [21]. Indeed, to avoid secondary headaches masquerading as misrecognition, clinicians should ask about the onset of each head pain individually; post-traumatic headache and medication overuse headaches, for example, may be easily separated out from their co-occurring primary headaches by inquiring carefully in regards to inciting incidents. If needed, a headache diary—one for each “kind” of headache—can be used to prospectively track phenotypes.

Our result also appears to offer the following insight for clinicians: misrecognition appears to occur frequently with primary headaches of short duration with known triggers. Consider the logically spurious diagnosis of SUNCT in the setting of combining primary sex headaches with PH. Therefore, clinicians should always ask about triggers. Indeed, the reason that triggers can play an important role is because they contain near pathognomonic characteristics that differentiate one diagnosis from all else. Similarly, the reason that some headache diagnoses are easily merged is because they have characteristics that are not pathognomonic. For example, our result suggests that most misrecognition arises from duration, which can be shared amongst many kinds of headaches.

### Limitations and Future Directions

A limitation of our project is that our dataset only includes primary headaches. A future direction would be to include both secondary headaches as well as facial pain. Although this project’s theorem can be applied to secondary headaches on a case-by-case basis (as demonstrated in Section 3), a comprehensive evaluation of all logically spurious diagnoses involving secondary headaches as well as facial pain is not part of this project and may be an important future direction.

This project also requires computational and empirical studies in the future to determine the frequency and occurrences of logically spurious diagnoses in real clinical settings. However, such studies on primary headaches may be limited by empirical constraints: consider the case where two clinicians disagree on a single case presentation when it comes to an illusionary/logically spurious diagnosis; it may be difficult to disentangle who has the correct answer. Therefore, a more objective alternate future direction may be to use an AI-powered training tool to study the issue of misrecognition: recently, Hakkinen et al. has demonstrated that simulated patients can be used in headache specialist training; assessing the frequency with which clinicians misjudge in simulated patients may overcome the limitations in empirical clinical studies [24,25,26]. In cases where secondary headaches are involved, future systematic surveys of patients in concussion and CSF leak clinics as well as mediation overuse headache patients in headache clinics, with known concurrent diagnoses of secondary headaches, are likely to be a fruitful direction of research.

It is important to note that discernment of co-occurring headache disorders assumes a level of patient self-reflection. Headache medicine is a specialty that relies on history taking. Yet, history relies on the assumptions that the patient knows his or her symptoms. We—as headache medicine practitioners—are therefore always assuming that patients possess the ability and the know-how, subjectively, to separate different headaches. At the very least, we assume that when prompted with questions of different headaches, patients can discern the differences even if they themselves may not know the precise diagnosis. In real life this is not always possible: examples include patients with dementia, autism, post-traumatic disorder, or psychosis. Since we are always diagnosing patients’ headache disorders through their own perception, a kind of Lacanian méconnaissance is to some degree unavoidable [27].

Furthermore, our encoding system also does not determine the existence or absence of each characteristic—the practitioner does. For example, our model does not determine whether a patient would satisfy the migraine criteria of having nausea during an attack if that patient is nauseated in only 1 out of 10 attacks. Therefore, the automated diagnosis component, if implemented in real-life settings, assumes thoughtful and accurate user implementation of ICHD criteria.

Although all negative characteristics are included in this study—for example, the *lack of* conjunctival injection is modeled by the prime number 283—the ubiquitous characteristic of “not attributed to another disorder” is not modeled in this paper. Doing so side-steps the logical contradiction native to ICHD3: consider the example where a specific headache phenotype satisfies migraine without aura as well as NDPH. If so, the criteria of “not attributed to another disorder” would raise the question of whether one should exclude both migraine without aura and NDPH or whether one should include one over the other. In clinical practice we are often not so pedantic as to tell our patients with NDPH of migraine phenotype (cf Robbins et al.) that they are undiagnosable and therefore untreatable [20]. Yet, taking ICHD3 at face value, this is a true logical impasse.

Lastly, in light of the creation/potential creation of the ICHD4, the intention of this paper is not to identify pedantic edge cases of the ICHD3. Rather, the goal is to not only show that logically spurious diagnosis can occur as a result of how criteria are written but also to identify the condition of possibility of such occurrences mathematically. As such, I believe the most important part of this paper to be that of the theorem and its application as well as the observation that misrecognition can be overcome most readily by careful history taking.

## 5. Conclusions

Our result shows that careful history taking is required to both identify and to avoid misdiagnosis in patients who suffer from multiple concurrent headache disorders. Furthermore, I proved that set/subset relationship between diagnostic criteria plays an important role in misrecognition. Finally, this manuscript also highlights a few important suggestions for future developments of classification criteria such as ICHD4:

1. Ensuring that no diagnostic criteria are simply subsets of other disorders.
2. Implementing rules or algorithms to prevent the erroneous assignment of diagnoses.
3. Determining and specifying instances where two or more diagnoses must be considered mutually exclusive.

## Figures and Tables

**Table 1:** Logically related headache characteristics.

Column 1	Column 2
1 min to 24 h with severe pain (2)	1 to 600 s (5), 15 min up to 4 h after waking (7), 15 to 180 min (11), 2 to 30 min (17), 30 min to 7 days (19), 21 to 14 days per month (3), 1 s to 2 h (47), hours to days (179), less than 48 h (211), severe (479), greater than 5 min (163)
1 to 14 days per month (3)	more than 10 days per month (269)
1 to 600 s (5)	1 min to 24 h with severe pain (2), 2 to 30 min (17), 1 s to 2 h (47), less than 48 h (211), up to a few seconds (557), greater than 5 min (163)
15 min up to 4 h after waking (7)	1 min to 24 h with severe pain (2), 15 to 180 min (11), 2 to 30 min (17), 30 min to 7 days (19), 1 s to 2 h (47), less than 48 h (211), up to 72 h with mild intensity (547), greater than 5 min (163)
15 to 180 min (11)	1 min to 24 h with severe pain (2), 15 min up to 4 h after waking (7), 2 to 30 min (17), 30 min to 7 days (19), 1 s to 2 h (47), less than 48 h (211), up to 72 h with mild intensity (547), greater than 5 min (163)
2 to 30 min (17)	1 min to 24 h with severe pain (2), 1 to 600 s (5), 15 to 180 min (11), less than 48 h (211), up to a few seconds (557), greater than 5 min (163)
30 min to 7 days (19)	1 min to 24 h with severe pain (2), 15 min up to 4 h after waking (7), 15 to 180 min (11), 4 to 72 h (23), 1 s to 2 h (47), less than 48 h (211), up to 72 h with mild intensity (547), greater than 5 min (163)
4 to 72 h (23)	1 min to 24 h with severe pain (2), 30 min to 7 days (19), less than 48 h (211), up to 72 h with mild intensity (547), greater than 5 min (163)
1 s to 2 h (47)	1 min to 24 h with severe pain (2), 1 to 600 s (5), 15 min up to 4 h after waking (7), 15 to 180 min (11), 2 to 30 min (17), 30 min to 7 days (19), less than 48 h (211), up to 72 h with mild intensity (547), up to a few seconds (557), greater than 5 min (163)
greater than 1 per day (137)	greater than 15 days per month (139), greater than 5 per day (167), greater than 8 days per month (173), more than 1 episode per day (251), more than 10 episodes (257), more than 10 days per month (269)
greater than 15 days per month (139)	every other day to 8 per day (107), more than 10 days per month (269)
greater than 2 episodes (149)	greater than 1 per day (137), greater than 20 episodes (151), greater than 5 episodes (157), more than 10 episodes (257)
greater than 20 episodes (151)	greater than 1 per day (137), greater than 2 episodes (149), greater than 5 episodes (157), more than 10 episodes (257)
greater than 5 episodes (157)	greater than 1 per day (137), greater than 2 episodes (149), greater than 20 episodes (151), more than 10 episodes (257)
greater than 5 min (163)	1 min to 24 h with severe pain (2), 1 to 600 s (5), 15 min up to 4 h after waking (7), 15 to 180 min (11), 2 to 30 min (17), 30 min to 7 days (19), 4 to 72 h (23), 1 s to 2 h (47), less than 48 h (211), up to 72 h with mild intensity (547)
greater than 5 per day (167)	greater than 1 per day (137), more than 1 episode per day (251)
greater than 8 days per month (173)	1 to 14 days per month (3), every other day to 8 per day (107), greater than 15 days per month (139), more than 10 days per month (269)
hours to days (179)	1 min to 24 h with severe pain (2), 15 min up to 4 h after waking (7), 15 to 180 min (11), 30 min to 7 days (19), 4 to 72 h (23),1 s to 2 h (47)
less than 48 h (211)	1 min to 24 h with severe pain (2), 1 to 600 s (5), 15 min up to 4 h after waking (7), 15 to 180 min (11), 2 to 30 min (17), 30 min to 7 days (19), 4 to 72 h (23), 1 s to 2 h (47), greater than 5 min (163), hours to days (179), up to 72 h with mild intensity (547), up to a few seconds (557)
mild to moderate pain (233)	1 min to 24 h with severe pain (2), moderate to severe (241), up to 72 h with mild intensity (547)
moderate to severe (241)	1 min to 24 h with severe pain (2), mild to moderate pain (233), severe (479)
more than 1 episode per day (251)	every other day to 8 per day (107), greater than 1 per day (137), greater than 15 days per month (139), greater than 1 per day (137), more than 10 days per month (269)
more than 10 episodes (257)	greater than 1 per day (137), greater than 2 episodes (149), greater than 20 episodes (151), greater than 5 episodes (157)
more than 10 days per month (269)	1 to 14 days per month (3), greater than 15 days per month (139), greater than 20 episodes (151), greater than 8 days per month (173)
severe (479)	1 min to 24 h with severe pain (2), moderate to severe pain (241)
up to 72 h with mild intensity (547)	1 to 600 s (5), 15 min up to 4 h after waking (7), 15 to 180 min (11), 2 to 30 min (17), 4 to 72 h (23), 1 s to 2 h (47), less than 48 h (211), mild to moderate pain (233)
up to a few seconds (557)	1 to 600 s (5), 1 s to 2 h (47), less than 48 h (211)

**Table 2:** Logical spurious diagnoses. An asterix (*) implies that the resultant spurious diagnosis is not caused artifactually by chronic migraine encoding.

Headache 1	Headache 2	Result of CM Encoding?	Diagnosed Headache 1	Diagnosed Headache 2	Diagnosed Headache 3	Diagnosed Headache 4	Diagnosed Headache 5
chronic migraine	chronic tension-type headache	*	migraine without aura	chronic migraine	frequent tension-type headache	chronic tension-type headache	
chronic migraine	chronic tension-type headache		migraine with aura	chronic migraine	chronic tension-type headache		
chronic migraine	chronic tension-type headache	*	migraine with aura	chronic migraine	frequent tension-type headache	chronic tension-type headache	
chronic migraine	cluster headache		cluster headache	migraine with aura	chronic migraine		
chronic migraine	cluster headache		migraine without aura	cluster headache	chronic migraine		
cold-induced headache	chronic migraine		cold-induced headache	migraine with aura	chronic migraine		
cold-induced headache	chronic migraine		migraine without aura	cold-induced headache	chronic migraine		
compression headache	chronic migraine		compression headache	migraine with aura	chronic migraine		
compression headache	chronic migraine		migraine without aura	compression headache	chronic migraine		
cough headache	cluster headache	*	cluster headache	SUNCT/SUNA	cough headache		
cough headache	chronic migraine		migraine without aura	chronic migraine	cough headache		
cough headache	chronic migraine		migraine with aura	chronic migraine	cough headache		
exercise headache	cluster headache	*	exercise headache	cluster headache	SUNCT/SUNA		
exercise headache	chronic migraine		exercise headache	migraine with aura	chronic migraine		
exercise headache	chronic migraine		migraine without aura	exercise headache	chronic migraine		
frequent tension type headache	chronic migraine		migraine without aura	chronic migraine	frequent tension-type headache		
frequent tension type headache	chronic migraine		migraine with aura	chronic migraine	frequent tension-type headache		
hemicrania continua	chronic migraine		hemicrania continua	migraine with aura	chronic migraine		
hemicrania continua	chronic migraine		migraine without aura	hemicrania continua	chronic migraine		
hypnic headache	chronic tension-type headache	*	hypnic headache	frequent tension-type headache	chronic tension-type headache		
hypnic headache	chronic migraine		migraine without aura	chronic migraine	hypnic headache		
hypnic headache	chronic migraine		migraine with aura	chronic migraine	hypnic headache		
infrequent tension type headache	chronic migraine		migraine without aura	chronic migraine	infrequent tension-type headache	frequent tension-type headache	
infrequent tension type headache	chronic migraine		migraine with aura	chronic migraine	infrequent tension-type headache	frequent tension-type headache	
infrequent tension type headache	hypnic headache	*	hypnic headache	infrequent tension-type headache	frequent tension type headache		
migraine without aura	chronic migraine		migraine without aura	migraine with aura	chronic migraine		
migraine without aura	hypnic headache	*	migraine without aura	chronic migraine	hypnic headache		
migraine with aura	chronic migraine		migraine without aura	migraine with aura	chronic migraine		
migraine with aura	hypnic headache	*	migraine with aura	chronic migraine	hypnic headache		
New Daily Persistent Headache	chronic migraine		migraine without aura	New Daily Persistent Headache	chronic migraine		
New Daily Persistent Headache	chronic migraine		New Daily Persistent Headache	migraine with aura	chronic migraine		
nummular headache	chronic migraine		migraine without aura	chronic migraine	nummular headache		
nummular headache	chronic migraine		migraine with aura	chronic migraine	nummular headache		
paroxysmal hemicrania	chronic tension-type headache	*	cluster headache	paroxysmal hemicrania	chronic tension-type headache		
paroxysmal hemicrania	chronic tension-type headache	*	cluster headache	paroxysmal hemicrania	SUNCT/SUNA	chronic tension-type headache	
paroxysmal hemicrania	cluster headache	*	cluster headache	paroxysmal hemicrania	SUNCT/SUNA		
paroxysmal hemicrania	chronic migraine	*	cluster headache	paroxysmal hemicrania	migraine with aura	chronic migraine	
paroxysmal hemicrania	chronic migraine	*	cluster headache	paroxysmal hemicrania	SUNCT/SUNA	migraine with aura	chronic migraine
paroxysmal hemicrania	chronic migraine	*	migraine without aura	cluster headache	paroxysmal hemicrania	chronic migraine	
paroxysmal hemicrania	chronic migraine	*	migraine without aura	cluster headache	paroxysmal hemicrania	SUNCT/SUNA	chronic migraine
paroxysmal hemicrania	cold-induced headache	*	cold-induced headache	paroxysmal hemicrania	SUNCT/SUNA		
paroxysmal hemicrania	compression headache	*	compression headache	paroxysmal hemicrania	SUNCT/SUNA		
paroxysmal hemicrania	cough headache	*	paroxysmal hemicrania	SUNCT/SUNA	cough headache		
paroxysmal hemicrania	exercise headache	*	exercise headache	paroxysmal hemicrania	SUNCT/SUNA		
paroxysmal hemicrania	frequent tension-type headache	*	paroxysmal hemicrania	SUNCT/SUNA	frequent tension-type headache		
paroxysmal hemicrania	hemicrania continua	*	paroxysmal hemicrania	SUNCT/SUNA	hemicrania continua		
paroxysmal hemicrania	hypnic headache	*	paroxysmal hemicrania	SUNCT/SUNA	hypnic headache		
paroxysmal hemicrania	infrequent tension-type headache	*	paroxysmal hemicrania	SUNCT/SUNA	infrequent tension-type headache		
paroxysmal hemicrania	migraine without aura	*	migraine without aura	paroxysmal hemicrania	SUNCT/SUNA		
paroxysmal hemicrania	migraine with aura	*	paroxysmal hemicrania	SUNCT/SUNA	migraine with aura		
paroxysmal hemicrania	New Daily Persistent Headache	*	New Daily Persistent Headache	paroxysmal hemicrania	hemicrania continua		
paroxysmal hemicrania	New Daily Persistent Headache	*	New Daily Persistent Headache	paroxysmal hemicrania	SUNCT/SUNA	hemicrania continua	
paroxysmal hemicrania	nummular headache	*	paroxysmal hemicrania	SUNCT/SUNA	nummular headache		
primary sex headache	cluster headache	*	cluster headache	SUNCT/SUNA	primary sex headache		
primary sex headache	chronic migraine		migraine without aura	chronic migraine	primary sex headache		
primary sex headache	chronic migraine		migraine with aura	chronic migraine	primary sex headache		
primary sex headache	paroxysmal hemicrania	*	paroxysmal hemicrania	SUNCT/SUNA	primary sex headache		
primary stabbing headache	cluster headache	*	cluster headache	SUNCT/SUNA	primary stabbing headache		
primary stabbing headache	chronic migraine		migraine without aura	chronic migraine	primary stabbing headache		
primary stabbing headache	chronic migraine		migraine with aura	chronic migraine	primary stabbing headache		
primary stabbing headache	paroxysmal hemicrania	*	paroxysmal hemicrania	SUNCT/SUNA	primary stabbing headache		
SUNCT/SUNA	chronic tension-type headache	*	cluster headache	SUNCT/SUNA	chronic tension-type headache		
SUNCT/SUNA	chronic migraine		migraine without aura	SUNCT/SUNA	chronic migraine		
SUNCT/SUNA	chronic migraine		SUNCT/SUNA	migraine with aura	chronic migraine		
SUNCT/SUNA	hemicrania continua	*	paroxysmal hemicrania	SUNCT/SUNA	hemicrania continua		
thunderclap headache	cluster headache	*	thunderclap headache	cluster headache	SUNCT/SUNA		
thunderclap headache	chronic migraine		migraine without aura	thunderclap headache	chronic migraine		
thunderclap headache	chronic migraine		thunderclap headache	migraine with aura	chronic migraine		
thunderclap headache	paroxysmal hemicrania	*	thunderclap headache	paroxysmal hemicrania	SUNCT/SUNA		
traction headache	chronic migraine		migraine without aura	traction headache	chronic migraine		
traction headache	chronic migraine		traction headache	migraine with aura	chronic migraine		
traction headache	paroxysmal hemicrania	*	traction headache	paroxysmal hemicrania	SUNCT/SUNA		

**Table 3:** Nontrivial logical spurious diagnoses.

Headache 1	Headache 2	Diagnosed Headache 1	Diagnosed Headache 2	Diagnosed Headache 3	Diagnosed Headache 4	Diagnosed Headache 5
cough headache	cluster headache	cluster headache	SUNCT/SUNA	cough headache		
exercise headache	cluster headache	exercise headache	cluster headache	SUNCT/SUNA		
hypnic headache	chronic tension-type headache	hypnic headache	frequent tension-type headache	chronic tension-type headache		
infrequent tension-type headache	hypnic headache	hypnic headache	infrequent tension-type headache	frequent tension-type headache		
migraine without aura	hypnic headache	migraine without aura	chronic migraine	hypnic headache		
migraine with aura	hypnic headache	migraine with aura	chronic migraine	hypnic headache		
paroxysmal hemicrania	chronic tension-type headache	cluster headache	paroxysmal hemicrania	chronic tension-type headache		
paroxysmal hemicrania	chronic tension-type headache	cluster headache	paroxysmal hemicrania	SUNCT/SUNA	chronic tension-type headache	
paroxysmal hemicrania	cluster headache	cluster headache	paroxysmal hemicrania	SUNCT/SUNA		
paroxysmal hemicrania	chronic migraine	cluster headache	paroxysmal hemicrania	migraine with aura	chronic migraine	
paroxysmal hemicrania	chronic migraine	cluster headache	paroxysmal hemicrania	SUNCT/SUNA	migraine with aura	chronic migraine
paroxysmal hemicrania	chronic migraine	migraine without aura	cluster headache	paroxysmal hemicrania	chronic migraine	
paroxysmal hemicrania	chronic migraine	migraine without aura	cluster headache	paroxysmal hemicrania	SUNCT/SUNA	chronic migraine
paroxysmal hemicrania	cold-induced headache	cold-induced headache	paroxysmal hemicrania	SUNCT/SUNA		
paroxysmal hemicrania	compression headache	compression headache	paroxysmal hemicrania	SUNCT/SUNA		
paroxysmal hemicrania	cough headache	paroxysmal hemicrania	SUNCT/SUNA	cough headache		
paroxysmal hemicrania	exercise headache	exercise headache	paroxysmal hemicrania	SUNCT/SUNA		
paroxysmal hemicrania	frequent tension-type headache	paroxysmal hemicrania	SUNCT/SUNA	frequent tension-type headache		
paroxysmal hemicrania	hemicrania continua	paroxysmal hemicrania	SUNCT/SUNA	hemicrania continua		
paroxysmal hemicrania	hypnic headache	paroxysmal hemicrania	SUNCT/SUNA	hypnic headache		
paroxysmal hemicrania	infrequent tension-type headache	paroxysmal hemicrania	SUNCT/SUNA	infrequent tension-type headache		
paroxysmal hemicrania	migraine without aura	migraine without aura	paroxysmal hemicrania	SUNCT/SUNA		
paroxysmal hemicrania	migraine with aura	paroxysmal hemicrania	SUNCT/SUNA	migraine with aura		
paroxysmal hemicrania	New Daily Persistent Headache	New Daily Persistent Headache	paroxysmal hemicrania	hemicrania continua		
paroxysmal hemicrania	New Daily Persistent Headache	New Daily Persistent Headache	paroxysmal hemicrania	SUNCT/SUNA	hemicrania continua	
paroxysmal hemicrania	nummular headache	paroxysmal hemicrania	SUNCT/SUNA	nummular headache		
primary sex headache	cluster headache	cluster headache	SUNCT/SUNA	primary sex headache		
primary sex headache	paroxysmal hemicrania	paroxysmal hemicrania	SUNCT/SUNA	primary sex headache		
primary stabbing headache	cluster headache	cluster headache	SUNCT/SUNA	primary stabbing headache		
primary stabbing headache	paroxysmal hemicrania	paroxysmal hemicrania	SUNCT/SUNA	primary stabbing headache		
SUNCT/SUNA	chronic tension-type headache	cluster headache	SUNCT/SUNA	chronic tension-type headache		
SUNCT/SUNA	hemicrania continua	paroxysmal hemicrania	SUNCT/SUNA	hemicrania continua		
thunderclap headache	cluster headache	thunderclap headache	cluster headache	SUNCT/SUNA		
thunderclap headache	paroxysmal hemicrania	thunderclap headache	paroxysmal hemicrania	SUNCT/SUNA		
traction headache	paroxysmal hemicrania	traction headache	paroxysmal hemicrania	SUNCT/SUNA		

**Table 4:** Nontrivial pairings containing migraine and tension-type headaches as well as potential ways to identify this. Example of each is in Supplementary File S2.

Headache Combination	Logically Spurious Diagnosis	Potential Identification Technique
Hypnic headache/chronic tension-type headache	frequent tension-type headache	Clarify how many episodes in a month are truly triggered by sleep. (Supplementary File S2 Example 1)
infrequent tension-type headache/hypnic headache	frequent tension-type headache	Clarify how many episodes in a month are truly triggered by sleep. (Supplementary File S2 Example 2)
migraine without aura/hypnic headache	chronic migraine	Clarify how many episodes in a month are truly triggered by sleep. (Supplementary File S2 Example 3)
migraine with aura/hypnic headache	chronic migraine	Clarify how many episodes in a month are truly triggered by sleep. (Supplementary File S2 Example 4)
paroxysmal hemicrania/chronic tension-type headache	Cluster headache	Tracking durations and frequency of attack that have TAC features, as opposed to duration of all attacks, may help clarify this. (Supplementary File S2 Example 5)
paroxysmal hemicrania/chronic tension-type headache	SUNCT/SUNA and cluster headache	Consider indomethacin for cluster and SUNCT/SUNA simply to empirically rule out these misrecognitions. (Supplementary File S2 Example 6)
paroxysmal hemicrania/chronic migraine	Cluster headache	Consider indomethacin for cluster simply to empirically rule out these misrecognitions. (Supplementary File S2 Example 7)
paroxysmal hemicrania/chronic migraine	Cluster headache and SUNCT/SUNA	Consider indomethacin for cluster or SUNA/SUNCT simply to empirically rule out these misrecognitions. (Supplementary File S2 Example 8)
paroxysmal hemicrania/chronic migraine	Cluster headache	Consider indomethacin for cluster simply to empirically rule out these misrecognitions. (Supplementary File S2 Example 9)
paroxysmal hemicrania/chronic migraine	Cluster headache and SUNCT/SUNA	Consider indomethacin for cluster or SUNA/SUNCT simply to empirically rule out these misrecognitions. (Supplementary File S2 Example 10)
paroxysmal hemicrania/frequent tension-type headache	SUNCT/SUNA	Consider indomethacin for SUNA/SUNCT simply to empirically rule out these misrecognitions. (Supplementary File S2 Example 11)
paroxysmal hemicrania/infrequent tension-type headache	SUNCT/SUNA	Consider indomethacin for SUNA/SUNCT simply to empirically rule out these misrecognitions. (Supplementary File S2 Example 12)
paroxysmal hemicrania/migraine without aura	SUNCT/SUNA	(Supplementary File S2 Example 13)
paroxysmal hemicrania/migraine with aura	SUNCT/SUNA	Consider indomethacin for SUNA/SUNCT simply to empirically rule out these misrecognitions. (Supplementary File S2 Example 14)
SUNCT/SUNA/chronic tension-type headache	cluster headache	Tracking durations and frequency of attack that have TAC features, as opposed to duration of all attacks, may help clarify this. (Supplementary File S2 Example 15)

## Data Availability

Source code and input files are provided as Supplementary Materials.

## Supplementary Materials

The following supporting information can be downloaded at: https://www.mdpi.com/article/10.3390/diagnostics16193231/s1, Supplementary File S1: dictionary Criteria; Supplementary File S2: examples of Table 4; Supplementary File S3: ichd3 Implications; Supplementary File S4: meconaissance.hs; Supplementary File S5: prime Encoding ICHD3; Supplementary File S6: Table S1: Prime Number Assignments for Headache Characteristics; Supplementary File S7: Table S2: Nontrivial logical spurious diagnoses; Supplementary File S8: Table S3: Nontrivial pairing containing migraine and tension type headaches as well as potential ways to identify this. Example of each is in Supplementary File S2.

## Abbreviations

The following abbreviations are used in this manuscript:

ICHD3	International classification of headache disorder, 3rd Edition
SUNCT	short lasting unilateral neuralgiform headaches
CM	chronic migraine
MwA	migraine with aura
NDPH	new daily persistent headache
HC	hemicrania continua
PH	paroxysmal hemicrania
MOH	medication overuse headache
PTH	posttraumatic headache
TTH	tension-type headache
CH	cluster headache

## References

[1] Headache Classification Committee of the International Headache Society (IHS) (2018). The International Classification of Headache Disorders, 3rd edition. Cephalalgia.

[2] Zhang P. (2023). Which headache disorders can be diagnosed concurrently? An analysis of ICHD3 criteria using prime encoding system. Front. Neurol..

[3] Zhang P. (2022). Disease Diagnosis and Classification as Prime Number Representations. hal-03723482. Lyon, France. https://hal.science/hal-03723482/.

[4] Gödel K. (1931). Über formal unentscheidbare Sätze der Principia Mathematica und verwandter Systeme I. Monatshefte für Math. Und Phys..

[5] Zhang P., Cheng R. (2026). Matrix and graphical representation of the primary headache syndromes in the International Classification of Headache Disorders (ICHD3): A basis for automated diagnosis and analysis of criteria. Front. Neurol..

[6] Moskatel L.S., Ogunlaja O., Zhang N. (2025). Prevalence, demographics, comorbidities, and treatment patterns of patients with the trigeminal autonomic cephalalgias: A retrospective analysis of United States electronic health records. BMC Neurol..

[7] Cohen F., Brooks C.V., Sun D., Buse D.C., Reed M.L., Fanning K.M., Lipton R.B. (2024). Prevalence and burden of migraine in the United States: A systematic review. Headache.

[8] Stovner L.J., Hagen K., Linde M., Steiner T.J. (2022). The global prevalence of headache: An update, with analysis of the influences of methodological factors on prevalence estimates. J. Headache Pain.

[9] Waliszewska-Prosół M., Straburzyński M., Czapińska-Ciepiela E.K., Nowaczewska M., Gryglas-Dworak A., Budrewicz S. (2023). Migraine symptoms, healthcare resources utilization and disease burden in a large Polish migraine cohort: Results from ‘Migraine in Poland’-a nationwide cross-sectional survey. J. Headache Pain.

[10] Stone J., Carson A., Duncan R., Roberts R., Warlow C., Hibberd C., Coleman R., Cull R., Murray G., Pelosi A. (2010). Who is referred to neurology clinics?—The diagnoses made in 3781 new patients. Clin. Neurol. Neurosurg..

[11] Tana C., Kodounis M., Ornello R., Raffaelli B., Messina R., Wells-Gatnik W., Waliszewska-Prosół M., Sacco S., Onan D., Martelletti P. (2026). Geriatric Migraine, Geroscience, and Sustainable Development Goals: Bridging Clinical Complexity and Public Health Priorities. J. Clin. Med..

[12] Evans R.W. (2015). Migraine mimics. Headache.

[13] Armand C.E., Masters-Israilov A., Lipton R.B. Migraine Mimcs: Are We Underdiagnosing Migraine? Practical Neurology, May 2019. https://practicalneurology.com/diseases-diagnoses/headache-pain/migraine-mimics/31510/.

[14] Angus-Leppan H. (2013). Migraine: Mimics, borderlands and chameleons. Pract. Neurol..

[15] Rossi P., Faroni J., Tassorelli C., Nappi G. (2009). Diagnostic delay and suboptimal management in a referral population with hemicrania continua. Headache.

[16] Peng K.P., Schellong M., May A. (2022). Aura in trigeminal autonomic cephalalgia is probably mediated by comorbid migraine with aura. Cephalalgia.

[17] Silberstein S.D., Niknam R., Rozen T.D., Young W.B. (2000). Cluster headache with aura. Neurology.

[18] Pareja J.A., Sjaastad O. (2010). Primary stabbing headache. Handb. Clin. Neurol..

[19] Braca S., Gimson A., Goadsby P.J. (2025). Primary stabbing headache in a tertiary headache centre. J. Headache Pain.

[20] Robbins M.S., Grosberg B.M., Napchan U., Crystal S.C., Lipton R.B. (2010). Clinical and prognostic subforms of new daily-persistent headache. Neurology.

[21] Friedman D.I. (2024). Approach to the Patient With Headache. Continuum.

[22] Mehta D., Cheema S., Glover S., Qureshi A.M., Davagnanam I., Kamourieh S., Sayal P., Toma A., Lagrata S., Joy C. (2025). Defining the typical characteristics of orthostatic headache in patients with spontaneous intracranial hypotension. Cephalalgia.

[23] Diener H.C., Kropp P., Dresler T., Evers S., Förderreuther S., Gaul C., Holle-Lee D., May A., Niederberger U., Moll S. (2022). Management of medication overuse (MO) and medication overuse headache (MOH) S1 guideline. Neurol. Res. Pract..

[24] Murinova N., Hakkinen I., Krashin D., Dave J., Zhang P., Lu M., Cuneo A., Sullivan H., Hakkinen M. (2026). Can AI-generated simulated patients be a tool to empower education in headache medicine: A pilot study (P9-15.002). Neurology.

[25] Murinova N., Hakkinen I., Krashin D., Dave J., Zhang P., Lu M., Cuneo A., Sullivan H., Hakkinen M. (2026). AI-generated simulated patients as a tool to enhance primary headache disorder treatment education: A pilot study. Headache.

[26] Jacobs C., Johnson H., Tan N., Brownlie K., Joiner R., Thompson T. (2025). Application of AI Communication Training Tools in Medical Undergraduate Education: Mixed Methods Feasibility Study Within a Primary Care Context. JMIR Med. Educ..

[27] Lacan J., Miller J.A. (1977). The Four Fundamental Concepts of Psycho-Analysis.

