# Supplementary material for: A Computational Analysis of Diagnostic Misrecognition in Co-Occurring Primary Headache Disorders: Prime Encoding of ICHD3 Criteria Reveals 72 Potentially Spurious Diagnoses
Source: Diagnostics (Basel). 2026 Oct 7;16(19):3231. doi: 10.3390/diagnostics16193231 (PMC13663992; doi:10.3390/diagnostics16193231)
Supplement: Supplementary file 1 [file diagnostics-16-03231-s001.zip › Supplementary File S2. examplesOfTable4.pdf]

1. Example of hypnic headache and chronic tension type headache (cTTH) leading to an illusion of frequent tension type headaches (fTTH).
    a. Example phenotypic pairings: Hypnic headache which occurs for less than 15 days per month but concurrent chronic tension type headache which occurs for more than 15 days per month. The misrecognition occurs when episodes of TTH are attributed to hypnic headache and so decrease the perceived frequency of TTH.
    b. Comments: The importance of this pairing is that one is led to believe that the tension type headache is less debilitating than it truly is. The way to identify this is to clarify how many episodes, potentially via headache diary, in a month is truly triggered by sleep.
2. Example of infrequent tension type headache (iTTH) and hypnic headache leading to illusion of fTTH.
    a. Example phenotypic pairings: Hypnic headache occurring for more than 10 days per month (as dictated by the ICHD3) but the iTTH occurring for > 5 days for 1 month. (Although the average is still less than 10 episodes per year.) the misrecognition arises when combining the frequency of hypnic headache and also the incidentally high 1 month of TTH.
    b. Comments: The problem here is that it makes TTH worse than it really is, thereby potentially triggering a prevention medication for the disease when it is not necessary. The way to identify this is to clarify how many episodes, potentially via headache diary, in a month is truly triggered by sleep.
3. Example of migraine without aura and hypnic headache leading to illusion of chronic migraine (CM):
    a. Example phenotypic pairings: Hypnic headache occurring for more than 15 days per month in the setting of migraine without aura occurring for less than 15 days per month. The illusion occurs when one attributes the occurrence of hypnic headache as migraine in terms of frequency.
    b. Comments: The treatment for hypnic headache is of course different than chronic migraine. This misrecognition changes management. Notice that this version is for migraine without aura; a similar one for migraine with aura exists. A solution is to Clarify how many episodes in a month is truly triggered by sleep.
4. Example of migraine with aura and hypnic headache leading to illusion of CM:
    a. Example phenotypic pairings: Similar to example 3.
    b. Comments: The treatment for hypnic headache is of course different than chronic migraine. This misrecognition changes management. Notice that this version is for migraine with aura; a similar one for migraine without aura exists. Clarify how many episodes in a month is truly triggered by sleep.

5. Example of paroxysmal hemicrania (PH) and cTTH leading to illusion of cluster headache (CH):
    a. Example phenotypic pairings: PH can be mistaken for cluster if the duration is between 15 to 30 minutes and/or if Indomethacin trial is not done. This occurs when the cTTH frequency occurs
    b. Comments: The misrecognition of cluster can significantly change management. Specifically, it may distract the clinician from an indomethacin trial. The fact that PH can be mistaken for cluster occurs even without adding on another diagnosis but TTH can cause the illusion of attacks that are more than 30 minutes. Tracking durations and frequency of attack that have TAC features, as opposed to duration of all attacks, may help clarify this.
6. Example of PH and cTTH leading to illusion of SUNCT/SUNA and cluster:
    a. Example phenotypic pairings: PH can in general be misrecognized as SUNCT if duration is between 2 minutes to 10 minutes (i.e. 600 seconds) or cluster if attacks are between 15 minutes to 30 mins and without an indomethacin trial; cTTH helps to satisfy the 20 attacks criteria for SUNCT and also every other day frequency for cluster since it is by definition > 15 days per month for > 3 months.
    b. Comments: This misrecognition can change management. Clinically, that SUNCT/SUNA may have baseline pain further allows the existence of cTTH in the setting of PH to distort the diagnosis. This builds the case of trying indomethacin for SUNCT/SUNA not because indomethacin has any evidence for the disease but simply to empirically rule out these misrecognitions. Similar argument can be made for cluster.
7. Example of PH and CM (with aura) leading to illusion of cluster:
    a. Example phenotypic pairings: CM is defined to be > 15 days per month. This frequency, together with the overlap between PH and cluster criteria when it comes to duration, allows cluster to be potentially satisfied as an illusionary diagnosis.
    b. Comments: The misrecognition of cluster can significantly change management. Specifically, it may distract the clinician from an indomethacin trial. Similar to prior cases, this supports indomethacin trial for cluster not because indomethacin has any evidence for the disease but simply to empirically rule out these misrecognitions.
8. Example of PH and CM (with aura) leading to illusions of SUNCT/SUNA and also cluster:
    a. Example phenotypic pairings: See example 7 and also example 14. The migraine without aura version is in example 10.

    b. Comments: This supports indomethacin trial for cluster/SUNCT/SUNA not because indomethacin has any evidence for the disease but simply to empirically rule out these misrecognitions.
9. Example of PH and CM (without aura) leading to illusion of cluster:
    a. Example phenotypic pairings: see example 7.
    b. Comments: See example 7
10. Example of PH and CM (without aura) leading to illusion of SUNCT/SUNA and also cluster:
    a. Example phenotypic pairings: see example 8.
    b. Comments: see example 8.
11. Example of PH and fTTH leading to illusion of SUNCT/SUNA:
    a. Example phenotypic pairings: Similar to previous, PH between 2 to 10 minutes (600 seconds) can be mistaken for SUNCT/SUNA.
    b. Comments: This supports indomethacin trial for SUNCT/SUNA not because indomethacin has any evidence for the disease but simply to empirically rule out these misrecognitions.
12. Example of PH and iTTH leading to illusion of SUNCT/SUNA:
    a. Example phenotypic pairings: see example 11
    b. Comments: see example 11
13. Example of PH and migraine without aura leading to illusion of SUNCT/SUNA:
    a. Example phenotypic pairings: Similar rationale to example 11.
    b. Comments: See example 11.
14. Example of PH and migraine with aura leading to illusion of SUNCT/SUNA:
    a. Example phenotypic pairings: see example 13
    b. Comments: see example 13
15. Example of SUNCT/SUNA and cTTH leading to illusion of cluster headache:
    a. Example phenotypic pairings: consider a SUNCT/SUNA phenomenon in the setting of a chronic tension type headaches which has the duration of 2 hours and by definition > 15 days per month. This is possible since the duration criteria of cTTH is "hours to days", so 2 hours would satisfy this criteria.
    b. Comments: The misrecognition of cluster can significantly change management. Specifically, it may distract the clinician from starting lamotrigine, the more appropriate treatment for SUNCT/SUNA. Unlikely misrecognition involving PH, indomethacin trial cannot be used. In these cases it is best to track the duration of TAC episodes and not just any headache episodes.

Note that the example presented is not intend to be an exhaustive compilation.
