# Supplementary material for: A Computational Analysis of Diagnostic Misrecognition in Co-Occurring Primary Headache Disorders: Prime Encoding of ICHD3 Criteria Reveals 72 Potentially Spurious Diagnoses
Source: Diagnostics (Basel). 2026 Oct 7;16(19):3231. doi: 10.3390/diagnostics16193231 (PMC13663992; doi:10.3390/diagnostics16193231)
Supplement: Supplementary file 1 [file diagnostics-16-03231-s001.zip › Supplementary File S6. Table S1.pdf]

Table S1. Prime Number Assignments for Headache Characteristics.

2	1 minute to 24 hours with severe intensity
3	1 to 14 days per month
5	1 to 600 seconds
7	15 minutes up to four hours after waking
11	15 to 180 minutes
13	1â€“6 cm in diameter
17	2 to 30 minutes
19	30 min to 7 days in duration
23	4 to 72 hours
29	abrupt explosive intensity just before or with orgasm
31	aggravated by physical activity
37	at least one aura symptom is positive
41	at least one aura symptom is unilateral
43	at least one aura symptom spreads gradually over 5 minutes
47	between 1 second to 2 hours
53	bilateral location
59	brainstem aura
61	brought on by cold stimuli

67	brought on by exercise
71	brought on by sex
73	brought on within 1 hour of compression
79	brought on within 1 hour of traction
83	clearly remembered onset
89	conjunctival injection
97	constant
101	developing only during sleep and causing wakening
103	each individual aura symptom lasts 5-60 minutes
107	every other day to 8 per day
109	eyelid edema
113	fixed in size and shape
127	forehead and facial sweating
131	fully reversible
137	greater than 1 per day
139	greater than 15 days per month
149	greater than 2 episodes
151	greater than 20 episodes
157	greater than 5 episodes

163	greater than 5 minutes
167	greater than 5 per day
173	greater than 8 days per month
179	hours to days
181	increasing in intensity with increasing sexual excitement
191	indomethacin responsive
193	irregular frequency
197	lacrimation
199	less than 12 days per year
211	less than 48 hours
223	max within 1 minute
227	maximal at site of compression
229	maximal at site of traction
233	mild to moderate pain
239	miosis
241	moderate to severe
251	more than 1 episode per day
257	more than 10 episodes
263	more than 3 months

269	more than 10 days per month
271	motor aura
277	nasal congestion
281	nausea/vomiting
283	no conjunctival injection
293	no eyelid edema
307	no forehead and facial sweating
311	no lacrimation
313	no miosis
317	no nasal congestion
331	no nausea/vomiting
337	no orbital or supraorbital or temporal pain
347	no phonophobia
349	no photophobia
353	no ptosis
359	no restless
367	no rhinorrhea
373	nonpulsating
379	not aggrevated by activity

383	orbital or supraorbital or temporal pain
389	phonophobia
397	photophobia
401	provoke by cough
409	provoke by valsalva
419	ptosis
421	pulsating
431	relieve by triptan or ergot
433	resolve within 1 hour after removal of compression
439	resolve within 1 hour after removal of traction
443	resolve within 30 min after removal of cold
449	restless
457	retinal aura
461	rhinorrhea
463	round or elliptical
467	sensory aura
479	severe
487	sharply contoured
491	single or series of stabs

499	speech and/or language aura
503	sudden
509	the aura is accompanied or followed within 60 minutes by headache
521	two or more aura symptoms occur in succession
523	unilateral
541	unremitting within 24 hours
547	up to 72 hours with mild intensity
557	up to few seconds
563	visual aura

Note:
ALL negative criteria are explicitly encoded except for "not attributed to another disorder". The following are the pairings of positive and negative criterion:

- 89 – conjunctival injection vs. 283 – no conjunctival injection
- 109 – eyelid edema vs. 293 – no eyelid edema
- 127 – forehead and facial sweating vs. 307 – no forehead and facial sweating
- 197 – lacraimation vs. 311 – no lacrimation
- 239 – miosis vs. 313 – no miosis
- 277 – nasal congestion vs. 317 – no nasal congestion
- 281 – nausea/vomiting vs. 331 – no nausea/vomiting
- 383 – orbital or supraorbital or temporal pain vs. 337 – no orbital or supraorbital or temporal pain
- 389 – phonophobia vs. 347 – no phonophobia
- 397 – photophobia vs. 349 – no photophobia
- 419 – ptosis vs. 353 – no ptosis
- 449 restlessness vs. 359 – no restlessness
- 461 – rhinorrhea vs. 367 – no rhinorrhea
- 421 – pulsating vs. 373 – nonpulsating
- 31 – aggravated by physical activity vs. 379 – not aggravated by activity
