# Supplementary material for: A Computational Analysis of Diagnostic Misrecognition in Co-Occurring Primary Headache Disorders: Prime Encoding of ICHD3 Criteria Reveals 72 Potentially Spurious Diagnoses
Source: Diagnostics (Basel). 2026 Oct 7;16(19):3231. doi: 10.3390/diagnostics16193231 (PMC13663992; doi:10.3390/diagnostics16193231)
Supplement: Supplementary file 1 [file diagnostics-16-03231-s001.zip › Supplementary File S7. Table S2.pdf]

Table S2. Nontrivial logical spurious diagnoses.

Headache 1	Headache 2	Diagnosed Headache 1	Diagnosed Headache 2	Diagnosed Headache 3	Diagnosed Headache 4	Diagnosed Headache 5
cough	cluster	cluster	SUNCT/SUNA	cough		
exercise	cluster	exercise	cluster	SUNCT/SUNA		
hypnic	chronic tension type headache	hypnic	frequent tension type headache	chronic tension type headache		
infrequent tension type headache	hypnic	hypnic	infrequent tension type headache	frequent tension type headache		
migraine w/o aura	hypnic	migraine w/o aura	cm	hypnic		
mwa	hypnic	mwa	cm	hypnic		
ph	chronic tension type headache	cluster	ph	chronic tension type headache		
ph	chronic tension type headache	cluster	ph	SUNCT/SUNA	chronic tension type headache	
ph	cluster	cluster	ph	SUNCT/SUNA		
ph	cm	cluster	ph	mwa	cm	
ph	cm	cluster	ph	SUNCT/SUNA	mwa	cm
ph	cm	migraine w/o aura	cluster	ph	cm	
ph	cm	migraine w/o aura	cluster	ph	SUNCT/SUNA	cm
ph	coldHA	coldHA	ph	SUNCT/SUNA		
ph	compression	compression	ph	SUNCT/SUNA		
ph	cough	ph	SUNCT/SUNA	cough		
ph	exercise	exercise	ph	SUNCT/SUNA		
ph	frequent tension type headache	ph	SUNCT/SUNA	frequent tension type headache		
ph	hc	ph	SUNCT/SUNA	hc		
ph	hypnic	ph	SUNCT/SUNA	hypnic		
ph	infrequent tension type headache	ph	SUNCT/SUNA	infrequent tension type headache		
ph	migraine w/o aura	migraine w/o aura	ph	SUNCT/SUNA		
ph	mwa	ph	SUNCT/SUNA	mwa		
ph	ndph	ndph	ph	hc		
ph	ndph	ndph	ph	SUNCT/SUNA	hc	
ph	nummular	ph	SUNCT/SUNA	nummular		
sex	cluster	cluster	SUNCT/SUNA	sex		

sex	ph	ph	SUNCT/SUNA	sex
stabbing	cluster	cluster	SUNCT/SUNA	stabbing
stabbing	ph	ph	SUNCT/SUNA	stabbing
SUNCT/SUNA	chronic tension type headache	cluster	SUNCT/SUNA	chronic tension type headache
SUNCT/SUNA	hc	ph	SUNCT/SUNA	hc
thunderclap	cluster	thunderclap	cluster	SUNCT/SUNA
thunderclap	ph	thunderclap	ph	SUNCT/SUNA
traction	ph	traction	ph	SUNCT/SUNA
