# Supplementary material for: A Computational Analysis of Diagnostic Misrecognition in Co-Occurring Primary Headache Disorders: Prime Encoding of ICHD3 Criteria Reveals 72 Potentially Spurious Diagnoses
Source: Diagnostics (Basel). 2026 Oct 7;16(19):3231. doi: 10.3390/diagnostics16193231 (PMC13663992; doi:10.3390/diagnostics16193231)
Supplement: Supplementary file 1 [file diagnostics-16-03231-s001.zip › Supplementary File S8. Table S3.pdf]

Table S3. Nontrivial pairing containing migraine and tension type headaches as well as potential ways to identify this. Example of each is in Supplementary File S2.

Headache combination	Logically spurious diagnosis	Identification
Hypnic/ chronic tension type headache	frequent tension type headache	Clarify how many episodes in a month is truly triggered by sleep. (Appendix Example 1)
infrequent tension type headache/ hypnic	frequent tension type headache	Clarify how many episodes in a month is truly triggered by sleep. (Appendix Example 2)
migraine without aura/ hypnic	chronic migraine	Clarify how many episodes in a month is truly triggered by sleep. (Appendix Example 3)
migraine with aura/ hypnic	chronic migraine	Clarify how many episodes in a month is truly triggered by sleep. (Appendix Example 4)
paroxysmal hemicrania/ chronic tension type headache	cluster	Tracking durations and frequency of attack that have TAC features, as opposed to duration of all attacks, may help clarify this. (Appendix Example 5)
paroxysmal hemicrania/ chronic tension type headache	SUNCT/SUNA and clsuter	Consider indomethacin for cluster and SUNCT/SUNA simply to empirically rule out these misrecognitions. (Appendix Example 6)
paroxysmal hemicrania/ chronic migraine	cluster	Consider indomethacin for cluster simply to empirically rule out these misrecognitions. (Appendix Example 7)
paroxysmal hemicrania/ chronic migraine	Cluster and SUNCT/SUNA	Consider indomethacin for cluster or SUNA/SUNCT simply to empirically rule out these misrecognitions. (Appendix Example 8)
paroxysmal hemicrania/ chronic migraine	cluster	Consider indomethacin for cluster simply to empirically rule out these misrecognitions. (Appendix Example 9)
paroxysmal hemicrania/ chronic migraine	Cluster and SUNCT/SUNA	Consider indomethacin for cluster or SUNA/SUNCT simply to empirically rule out these misrecognitions. (Appendix Example 10)
paroxysmal hemicrania/ frequent tension type headache	SUNCT/SUNA	Consider indomethacin for SUNA/SUNCT simply to empirically rule out these misrecognitions. (Appendix Example 11)
paroxysmal hemicrania/ infrequent tension type headache	SUNCT/SUNA	Consider indomethacin for SUNA/SUNCT simply to empirically rule out these misrecognitions. (Appendix Example 12)
paroxysmal hemicrania/ migraine without aura	SUNCT/SUNA	(Appendix Example 13)
paroxysmal hemicrania/ migraine with aura	SUNCT/SUNA	Consider indomethacin for SUNA/SUNCT simply to empirically rule out these misrecognitions. (Appendix Example 14)

SUNCT/SUNA/ chronic tension type headache	clsuter	Tracking durations and frequency of attack that have TAC features, as opposed to duration of all attacks, may help clarify this. (Appendix Example 15)
